# Brown adipose tissue-derived extracellular vesicles regulate hepatocyte mitochondrial activity to alleviate high-fat diet-induced jawbone osteoporosis in mice

**DOI:** 10.3389/fendo.2025.1583408

**Published:** 2025-04-24

**Authors:** Kai Zhang, Sha Zhang, Guorong Deng, Guangxiang He, Yuan Yuan, Yu Fu, Yihan Liu, Zhen Gong, Liang Kong, Chenxi Zheng

**Affiliations:** ^1^ State Key Laboratory of Oral & Maxillofacial Reconstruction and Regeneration, National Clinical Research Center for Oral Diseases, Shaanxi International Joint Research Center for Oral Diseases, Center for Tissue Engineering, School of Stomatology, The Fourth Military Medical University, Xi’an, China; ^2^ State Key Laboratory of Oral & Maxillofacial Reconstruction and Regeneration, National Clinical Research Center for Oral Diseases, Shaanxi Clinical Research Center for Oral Diseases, Department of Oral and Maxillofacial Surgery, School of Stomatology, The Fourth Military Medical University, Xi’an, China; ^3^ College of Basic Medicine, Shaanxi Key Laboratory of Research on TCM Physical Constitution and Diseases Prevention and Treatment, Shaanxi University of Chinese Medicine, Xianyang, China; ^4^ Department of Traditional Chinese Medicine, The First Affiliated Hospital of Fourth Military Medical University, Xi’an, China; ^5^ Department of Critical Care Medicine, The Second Affiliated Hospital of Xi’an Jiaotong University, Xi’an, China; ^6^ The First Clinical Medical College, Shaanxi University of Chinese Medicine, Xianyang, China; ^7^ Department of Stomatology, The First Medical Center, Chinese Chinese PLA General Hospital, Beijing, China; ^8^ Analysis & Testing Laboratory for Life Sciences and Medicine of Fourth Military Medical University, Xi’an, China

**Keywords:** lipid metabolic disorder, jawbone osteoporosis, brown adipose tissue, extracellular vesicles, mitochondrial activity, osteogenic microenvironment

## Abstract

**Background:**

Lipid metabolic disorder (LMD) serves as a systemic driver of osteoporosis (OP), with jawbone osteoporosis (JOP) representing a clinically significant yet underexplored complication. Current clinical treatments for JOP remain suboptimal, highlighting the need for innovative approaches. The use of metabolic regulators represents a promising therapeutic strategy for OP management. While brown adipose tissue-derived extracellular vesicles (BEV) exhibit metabolic regulatory potential, their capacity to mitigate LMD-associated OP remains unclear.

**Methods:**

A high-fat diet (HFD)-induced LMD mouse model was established to identify the JOP phenotype through micro-computed tomography (micro-CT) and transcriptomic profiling. BEV isolation was optimized using liberase enzyme-enhanced differential centrifugation, with *in vivo* tracking confirming biodistribution. *In vitro*, BEV effects on hepatocytes were assessed with triglyceride (TG) content, free fatty acid (FFA) levels, and mitochondrial function. The additional benefits of BEV on the osteogenic microenvironment were evaluated *via* AML12/MC3T3-E1 indirect co-culture under high-lipid conditions. Dual therapeutic effects of BEV on LMD and JOP *in vivo* were validated through metabolic phenotyping, micro-CT and histomorphometry analysis.

**Results:**

Sixteen weeks of HFD successfully induced typical LMD and JOP manifestations in mice. Transcriptomic sequencing revealed downregulation of osteogenic-related genes concomitant with upregulation of lipid metabolism-associated genes in the jawbone of LMD mice. *In vivo* tracking showed the exogenous BEV predominantly accumulated in the liver rather than the jawbone. BEV treatment significantly reduced intracellular TG and FFA content in hepatocytes, while enhancing osteogenic activity of MC3T3-E1 cells through indirect co-culture. Mitochondrial analyses revealed that BEV effectively increased the proportion of active mitochondria, reduced reactive oxygen species (ROS) generation rate, and enhanced oxygen consumption rate (OCR) in hepatocytes. Biochemical assay and metabolic cage testing showed a lower systemic lipid content level along with improved fat utilization and thermogenesis capacity in BEV-treated mice. Micro-CT and immunofluorescence staining further confirm significant improvements in the jawbone of BEV-treated mice regarding bone volume fraction, trabecular number, trabecular thickness, trabecular separation, and RUNX2 expression.

**Conclusion:**

This study establishes LMD as a crucial driver factor in JOP and identifies BEV-mediated mitochondrial transferring in hepatocytes as a therapeutic strategy for LMD-related JOP.

## Introduction

Lipid metabolism disorder (LMD), characterized by systemic dysregulation of lipid homeostasis, represents a growing global health burden linked to complications, such as non-alcoholic fatty liver disease (NAFLD), insulin resistance, and cardiovascular diseases (1, 2). Hepatic dysfunction, as one of the core driving factors of LMD, has been confirmed to be closely associated with mitochondrial dysfunction. Mitochondrial dysfunction in lipid metabolism, characterized by abnormal synthesis and catabolism of lipids during energy production, may trigger ectopic lipid deposition, reactive oxygen species (ROS) accumulation, and a series of subsequent cellular injuries (3, 4). Regulation of hepatic mitochondrial function to improve metabolic function represents a promising strategy for the treatment of LMD and LMD-related diseases.

The jawbone, characterized by lifelong remodeling capacity, is one of the most metabolically active bone tissues, which keeps a structural and functional balance based on the metabolic microenvironment (5). Osteogenic-unfavorable microenvironment (*e.g.*, high lipid conditions) inhibits osteogenic process and leads to jawbone osteoporosis (JOP) (5–7). Additionally, studies showed a microenvironment with high-lipid concentration inducing the bone marrow stem cell to differentiate into adipocytes instead of osteogenic precursor cells, thereby suppressing the initiation of bone formation (8–10). Despite these findings, the relationship between LMD and JOP has received limited attention in *in vivo* studies. Furthermore, current treatments for JOP predominantly focus on symptomatic management of the jawbone, with few etiologic interventions being considered. It is also noteworthy that commonly used osteoporosis medications, such as bisphosphonates, have been associated with jawbone necrosis (11–13). Given these challenges, the development of a novel strategy with dual therapeutic effects on LMD and JOP represents an emerging and significant demand.

Adipose tissue in mammals can be classified into three types: white adipose tissue (WAT), brown adipose tissue (BAT), and beige adipose tissue (BeAT) (14). Among them, BAT is considered to have the strongest metabolic regulatory capacity. Characterized by the expression of uncoupling protein 1 (UCP1) in the mitochondrial inner membrane, BAT facilitates the rapid dissipation of excess energy as heat (15). This unique thermogenic capacity enables BAT to exert beneficial effects on a range of metabolic disorders. Recent studies have shown that BAT can regulate the metabolism of distant tissue through the secretion of extracellular vesicles (EVs) (16). With nanoscale, low immunogenicity and robust loading capacity, BAT-derived EVs (BEV) have shown great potential as a natural regulator in treating metabolic related disease (17, 18). Studies demonstrate that BEV ameliorating LMD-related diseases like hepatic steatosis and cardiomyopathy by transporting mitochondria-modulating miRNAs and thermogenic proteins (16–20). However, the regulatory function and mechanism of BEV in LMD-JOP remains unclear.

In this study, we established an LMD-JOP model on a high-fat diet (HFD)-induced mouse to investigate the manifestation and the transcriptomic profiling of jawbone under LMD conditions. BEV was isolated directly from the brown adipose tissue with liberase enzyme-enhanced differential centrifugation. Furthermore, the therapeutic effects of BEV on LMD and LMD-JOP were examined through the intravenous injection of BEV. A potential mechanism of BEV ameliorating LMD-JOP by modulating mitochondrial activity in hepatocytes was explored with *in vitro* and *in vivo* analysis. EVs isolated from white adipose tissue (WEV) were analyzed as negative control throughout this study. This study provides novel insights into the relationship between LMD and JOP, and it holds promise for the development of a novel therapeutic strategy based on BEV that modulates LMD and thereby improves JOP.

## Materials and methods

### Establishment of the HFD-induced JOP model in mice

Four-week-old male C57BL/6J mice were housed in well-ventilated conditions under a 12-hour light/12-hour dark cycle. Two groups were formed: the control group (Control) and the HFD, each consisting of 16 mice, resulting in a total of 32 mice. The Control group received a standard maintenance diet, whereas the HFD group was given a diet comprising 60% fat (FinnemoreTech, China). Both groups had unrestricted access to food and water, and chew sticks were provided in each cage to mitigate the risk of disusing JOP. The fat, liquid and lean ratio of the mice was recorded with a whole-body composition analyzer (Bruker LF90, Bruker, German). After 16 weeks of dietary intervention, the mice were euthanized through CO_2_ asphyxiation (KW-AL-G, NJKEWBIO, China) and placed on ice for tissue collection. The mandible samples were harvested as representative of the jawbone. Other tissues were subsequently processed or preserved according to the requirements for further experiments.

### Glucose tolerance test and insulin tolerance test

Blood samples were collected from the tail vein of mice and measured with a glucose meter (580, Yuwell, China) and matching blood glucose test strips. For GTT, mice were fasted for 18 hours and then intraperitoneally injected with glucose solution (ST1228-250g, Beyotime, China) at a dose of 1 g/kg. For ITT, mice were fasted for 6 hours and then intraperitoneally injected with insulin (P3376-100IU, Beyotime, China) at a dose of 0.4 IU/kg. Blood glucose levels were measured at 0, 15, 30, 60, 90, and 120 minutes after injection. The area under curve (AUC) of each sample was calculated to show the difference between groups.

### Triglyceride and total cholesterol measurements in serum and liver

The fresh blood was harvested from the orbital sinus of mice and centrifuged at 3,000 rpm for 15 minutes (4°C). The supernatant was collected and stored at -80°C for further use. For preparation of liver tissue samples, 100 mg fresh harvested liver tissue was mixed with 900 µL pure ethanol and smashed into homogenate, followed by a centrifugation at 2,500 rpm for 10 minutes (4°C) to collect the supernatant. For measurement, the content of TG in samples was measured according to the instructions of Triglyceride Assay Kit (A110-1-1, Nanjing Jiancheng Bioengineering Institute, China), and the content of TC was measured following the instructions of Total Cholesterol Assay Kit (A111-1-1, Nanjing Jiancheng Bioengineering Institute, China).

### Hematoxylin and eosin staining

After tissue collection, fresh jawbone, adipose, or liver tissue samples were fixed in 4% paraformaldehyde (PFA) (P0099, Beyotime, China) for 48 hours and then rinsed overnight in running water. For jawbone samples, decalcification was performed in 10% EDTA (HT114, HAT, China) solution for 15 days, with the solution changed every other day. Once decalcified, the jawbone samples were washed with PBS (G4202, Servicebio, China) and processed for paraffin (WGHB, Servicebio, China) embedding. Adipose and liver tissues were directly processed for paraffin embedding after fixation. Following the standard H&E staining procedure, samples were dehydrated in graded ethanol, cleared in xylene, and embedded in paraffin to produce 6-µm-thick sections. For jawbone samples, sections were taken along the long axis of the jawbone to display the sagittal plane of the molar root bifurcation area. After sectioning, slides were baked, dewaxed, and rehydrated through graded ethanol. H&E staining was performed according to the manufacturer’s instructions (C0105M, Beyotime, China), followed by mounting with neutral gum (C0173, Beyotime, China) and observation under a microscope.

### Immunofluorescence staining

After tissue collection, fresh jawbone or adipose tissue samples were fixed in 4% PFA for 48 hours and then rinsed overnight in running water. The samples were then immersed in 30% sucrose (P0149D, Beyotime, China) for dehydration at 4°C for 24 hours, followed by embedding in OCT compound (4583, Sakura Tissue-Tek, America). After freezing at -20°C, the samples were sectioned into 10-µm-thick cryosections using a cryostat microtome (CM1950, Leica, German), with the sectioning direction of jawbone tissue consistent with that of the H&E sections. The prepared tissue cryosections were treated with the normal protocol of immunofluorescence. Primary antibodies were diluted and applied as follows: for osteopontin (OPN) (1:200, sc-73631, Santa Cruz, America), runt-related transcription factor 2 (RUNX2) (1:200, #12556, Cell Signaling, America), and UCP1 (1:200, ab234430, Abcam, America). Secondary antibodies were diluted and applied as follows: for OPN (33212ES60, Yeasen, China), and for RUNX2 and UCP1 (33112ES60, Yeasen, China). DAPI-containing anti-fade immunofluorescence mounting medium (564907, BD Biosciences, America) was used before photographing with a confocal laser scanning microscope (FV1000, Olympus, Japan).

### Oil red O staining

Oil red O staining of liver tissue was performed on cryosections, prepared as described in the immunofluorescence staining. For staining, 10 mL of isopropanol (I434894, Aladdin, China) was mixed with 0.05 g of oil red O powder (O8010, Solarbio, China) and vortexed to dissolve completely to form the staining stock solution. This solution was kept at room temperature for 10 minutes and then filtered through a 220 nm filter (84301ES14, Yeasen, China). Nine milliliters of the filtered stock solution were mixed with 6 mL of water to form the working solution (60% isopropanol with oil red O). For staining, the freshly prepared oil red O working solution was added to cover the cryosections and incubated for 5 minutes. The sections were then slightly rinsed on running water for 1 minute, followed by immersion in 60% isopropanol (30 seconds) and hematoxylin (2 minutes) (ST2067, Beyotime, China). After washing and drying, the stained cryosections were mounted and observed under a microscope.

### Metabolic cage testing

A Comprehensive Lab Animal Monitoring System (CLAMS) (CLAMS-16, Columbus, China) was used as a metabolic cage to test the macroscopic metabolic activities of mice. The monitoring system provided a normal feeding and drinking environment with a light-dark cycle set from 8:30 to 20:30 (light) and from 20:30 to 8:30 the next day (dark). After weighing, the mice were placed individually into the monitoring cages and allowed to acclimate for 8~12 hours before continuous data collection for 72 hours. The core parameters measured were as follows: Respiratory exchange ratio (RER): defined as the ratio of CO_2_ production to O_2_ consumption; Heat production (kcal/kg/hr): the amount of heat produced by the mice per unit body weight and per unit time. The AUC of each sample was calculated to show the difference between groups.

### Micro-CT analysis of jawbone and femur

Freshly harvested jawbone and femur samples were dissected to remove surface muscle and fascia and then fixed in the scanning chamber of a small-animal micro-CT device (AX-2000, OY Detection Technology, China) for cone-beam scanning. The device parameters were set as follows: voltage at 80 kV, current at 70 µA, number of projections at 1440, integration time at 500 ms, frame rate at 2 frames, and resolution at 5 µm. After scanning, three-dimensional reconstruction was performed, focusing on the visualization of the jawbone in the mandibular root bifurcation area. The data were then imported into VG Studio Max 3.5 software for further analysis of the parameters.

### Jawbone transcriptome sequencing

The transcriptome sequencing was performed by OE Biotech Co., Ltd. (Shanghai, China) using fresh jawbone samples from the HFD and Control groups (n=5 per group). Total RNA was extracted using TRIzol reagent, and RNA quality was assessed using a NanoDrop 2000 spectrophotometer and Agilent 2100 Bioanalyzer. Transcriptome libraries were constructed with the VAHTS Universal V6 RNA-seq Library Prep Kit and sequenced on the Illumina Novaseq 6000 platform (150 bp paired-end reads). Raw reads were processed with fastp, aligned to the reference genome, and analyzed for differential expression using DESeq2 (criteria: adjusted *P* value < 0.05, |log_2_FC| > 1.00)). principal component analysis (PCA) was performed to assess sample replicates. Hierarchical clustering and functional enrichment analyses were conducted using R (v 3.2.0), and GSEA was performed to evaluate gene set enrichment.

### Isolation of EVs from adipose tissues

Adipose tissue-derived EVs were isolated according to our previously established protocol (21). Briefly, 1 g fresh and minced adipose tissue (brown or white) was placed in 15 mL centrifuge tubes containing 10 mL liberase enzyme solution (Sigma-Aldrich, USA, diluted at 1:1000) and incubated at 37°C for 30 minutes. Then, the samples were centrifuged at 800 g for 10 minutes to remove the cells in the pellet. The supernatant was collected and centrifuged at 2,000 g for 10 minutes to remove the cellular debris. After that, the supernatant was purified through a 450 nm filter, transferred to 1.5 mL EP tubes, and centrifuged at 16,000 g for 30 min at 4°C. The pellet was washed twice with PBS and the resulting adipose tissue-derived EVs were suspended with relevant solutions. All EVs were stored at 4°C and used for experiments within 24 h.

### Nanoparticle tracking analysis

NTA was performed using a PMX ZetaView instrument equipped with an EVs 520 nm model (Malvern, UK) to assess the size distribution and particle concentration of different EVs. Freshly prepared EV suspensions were diluted in sterile PBS at a ratio of 1:1000 to 1:20000 to achieve a final concentration of 1×10^8^ particles/mL. Particle analysis and report generation were conducted according to the manufacturer’s instructions.

### Transmission electron microscopy

After centrifugation, the EV samples were resuspended in an appropriate volume of PBS. A 5~6 μL aliquot of the sample suspension was directly applied to a copper grid and allowed to settle at room temperature for 5~10 minutes. The excess sample was blotted with filter paper. The grid was then negatively stained with uranyl acetate for 2~3 minutes, and the excess stain was removed with filter paper. The grid was washed three times with double-distilled water and air-dried. The morphology and structure of EVs were observed on a FEI Tecnai G2 Spirit Biotwin TEM (Thermo Fisher, USA).

### DiR labeling and *in vivo* tracking of EVs

Freshly prepared EV suspensions at an appropriate concentration were diluted and protein concentration was determined using the BCA (P0012, Beyotime, China) method. DiR dye (40757ES25, Yeasen, China) was added (diluted at 1:500) and incubated at room temperature in the dark for 10 minutes. After incubation, the sample was centrifuged twice at 16,000 g for 30 minutes and resuspended in PBS. Mice were depilated and accepted DiR-labeled EVs injection *via* the tail vein (10 μg of EVs for each mouse). *In vivo* tracking was performed using a live imaging system at 24 hours post-injection. Mice were euthanized, and the bright and fluorescence images of major organs were collected. Imaging parameters were as follows: IVIS Acquisition Control Panel Imaging Mode: Fluorescent; Exposure Time: Auto; Photograph: 0.20; Excitation Filter: 740 nm; Emission Filter: 790 nm.

### Uptake experiment of EVs

Freshly prepared EVs were centrifuged at 16,000 g for 30 minutes at 4°C, and the supernatant was discarded. 200 µL of Diluent C and 2 µL of PKH26 dye from the Red Fluorescent Cell Linker Mini Kit (Merck, German) were added to resuspend the pellet, and the volume was adjusted to 500 µL with Diluent C. After vertexing, the mixture was incubated at room temperature for 10 minutes. 500 µL of Diluent C containing 10% fetal bovine serum (A5670701, Gibco, America) was added to terminate the reaction, followed by centrifugation at 16,000 g for 30 minutes at 4°C. The pellet was resuspended in PBS. The protein concentration of the suspension was determined using the BCA method, and the remaining suspension was stored at 4°C. AML12 cells (AML12, Cellverse, China) were seeded into a 24-well plate at 2,000 cells per well and cultured in DMEM/F12 medium (11320033, Gibco, America), supplemented with 10% FBS, 1% double antibiotics (60162ES76, Yeasen, China), and 1% ITS liquid medium supplement (C0345, Beyotime, China). After 12 hours, labeled EVs were added to each well at a final concentration of 10 µg/mL. After 24 hours, the medium was replaced, and cells were fixed with 4% PFA and stained for F-actin (ab205, Abcam, China) and DAPI (C1005, Beyotime, China). The cells were then removed, mounted, and observed under a confocal microscope.

### Measurement of intracellular FFA and TG contents in AML12 cells

AML12 cells were digested, counted, and seeded into a 24-well culture plate at 20,000 cells per well. Palmitic acid (PA) (KC002, Kunchuang Tech, China) was added at a final concentration of 1.5 mM to simulate high lipid conditions. Four groups were set: Control, PA+PBS, PA+BEV, and PA+WEV, with three replicates per group. Except for the Control group, the final concentration of PA in each well was 0.15 mM, and the final concentration of EVs in the BEV and WEV groups was 10 µg/mL. The Control and PA+PBS groups did not contain EVs and were supplemented with an equal volume of PBS. After 24 hours of EVs treatment, the intracellular FFA and TG content of AML12 cells was measured using a free fatty acid assay kit (S0215S, Beyotime, China) and a triglyceride assay kit (S0219S, Beyotime, China).

### Indirect co-culture of AML12/MC3T3-E1 cells

AML12 cells were passaged into 10-cm dishes, and when reaching 80% confluence, the medium was changed to that containing different types of EVs (final concentration of 10 μg/mL) or PA (final concentration of 0.15 mM) according to the experimental groups. After 48 hours of culture, the supernatant (named Culture Medium A) was collected. Culture Medium A was sequentially centrifuged at 800 g for 10 minutes and 2,000 g for 10 minutes to remove cell debris, and then at 16,000 g for 30 minutes to remove EVs and impurities. It was further purified by filtration through a 220-nm filter. The supernatant was collected and mixed with α-MEM medium (G4555, Servicebio, China) at a 1:1 ratio (named Culture Medium B). β-glycerophosphate disodium (HY-126304, MedChemExpress, America) (final concentration of 10 mM), ascorbic acid (60374ES60, Yeasen, China) (final concentration of 50 µg/mL), and dexamethasone (40323ES60, Yeasen, China) (final concentration of 10 nM) were added to Culture Medium B to form the induction medium (named Culture Medium C). MC3T3-E1 cells (iCell-m031, Cellverse, China) were cultured in α-MEM medium under the same conditions as AML12 cells. When MC3T3-E1 cells reached a density of 50~70%, Culture Medium C was changed every other day for 7 days of osteogenic induction. Cells were then stained according to the instructions of the BCIP/NBT Alkaline Phosphatase (ALP) Color Development Kit (C3206, Beyotime, China). After staining, the proportion of the ALP-stained area was analyzed with ImageJ (1.54f, NIH, America).

### Quantitative real-time polymerase chain reaction analysis

After 7 days of osteogenic induction, MC3T3-E1 cells were washed twice and harvested using a cell scraper. Total RNA was extracted using TRIzol reagent (T9108, Takara Bio. Japan). cDNA was synthesized using the PrimeScript RT Master Mix (RR036A, Takara Bio. Japan). SYBR−Green PCR kit (RR420A, Takara Bio. Japan) was used as the fluorophore. The following conditions were used: 95˚C for 30 seconds (1 cycle), 95˚C for 5 seconds (40 cycles), and 60˚C for 30 seconds (40 cycles). The data were processed using the 2^−ΔΔcq^ method and the relative expression levels were calculated using U6 mRNA as an internal reference. The sequences of the primers (Tsingke biotech, China) were the following:

*Runx2* forward: 5’-ATGCTTCATTCGCCTCACAAA-3’ and reverse: 5’-GCACTCACTGACTCGGTTGG-3’.

*β-actin* forward: 5’-GTGACGTTGACATCCGTAAAGA-3’ and reverse: 5’-GCCGGACTCATCGTACTCC-3’.

### Flow cytometry detection of JC-1 and ROS

AML12 cells were divided into four groups: Control, PA+PBS, PA+BEV, and PA+WEV (three replicates per group). Except for the Control group, PA was added to a final concentration of 0.15 mM, and EVs were added to a final concentration of 10 µg/mL in the BEV and WEV groups. After 24 hours of EV treatment, mitochondria were isolated using the Cell Mitochondria Isolation kit (C3601, Beyotime, China). The proportion of active mitochondria was assessed using the JC-1 kit (C2006, Beyotime, China), and the ROS generation rate was measured using a kit (S0035S, Beyotime, China), following the manufacturer’s instructions. Flow cytometry was conducted on the Novocyte platform (2040R, Agilent Technologies, America) for analysis.

### Oxygen consumption rate detection

AML12 cells were digested, counted, and seeded into a 24-well Seahorse plate at 20,000 cells per well (300 µL per well) with four replicates per group. The Seahorse machine was preheated to 37°C, and probes were hydrated with XF calibration solution overnight. For detection, the XF base medium was prepared with substrates (pH 7.4) and kept in a 37°C water bath. Cells were checked under a microscope, and the medium was replaced with an assay solution before incubation at 37°C for 1 hour. Solutions of 1 µM oligomycin (HY-N6782, MedChemExpress, America), FCCP (HY-100410, MedChemExpress, America), and rotenone (HY-B1756, MedChemExpress, America) were prepared to use. The test plate and utility plate were loaded into instruments (Seahorse XF, Agilent Technologies, America) for automated calibration and detection. The data was analyzed using software following the manufacturer’s instructions.

### Observation of the therapeutic effects of BEV

Four-week-old male C57BL/6J mice were randomly divided into four groups (n = 8 per group) after acclimatization. One group was fed a normal diet and served as the Control group. The other three groups were fed an HFD to establish an LMD model. Starting from week 5 of HFD induction, exogenous adipose tissue EVs were administered *via* tail vein injection at a dose of 150 µg per mouse. The groups were designated as Control, HFD+PBS, HFD+BEV, and HFD+WEV. Injections were performed once a week for 12 weeks. Micro-CT, metabolic cage testing, and tissue sectioning were performed as described previously.

### Statistical analysis and schematics

All the data were presented as mean ± standard deviation (SD). Statistical and graph analysis was performed using GraphPad Prism 10.1.2 (GraphPad Software, America). For multiple group comparisons, significance was assessed by one-way analysis of variation (ANOVA) with Tukey’s *post hoc* test. Values of *P* < 0.05 were considered statistically significant. All schematics were drawn using Biorender with permission.

## Results

### HFD induces LMD in mice

**Figure 1:**
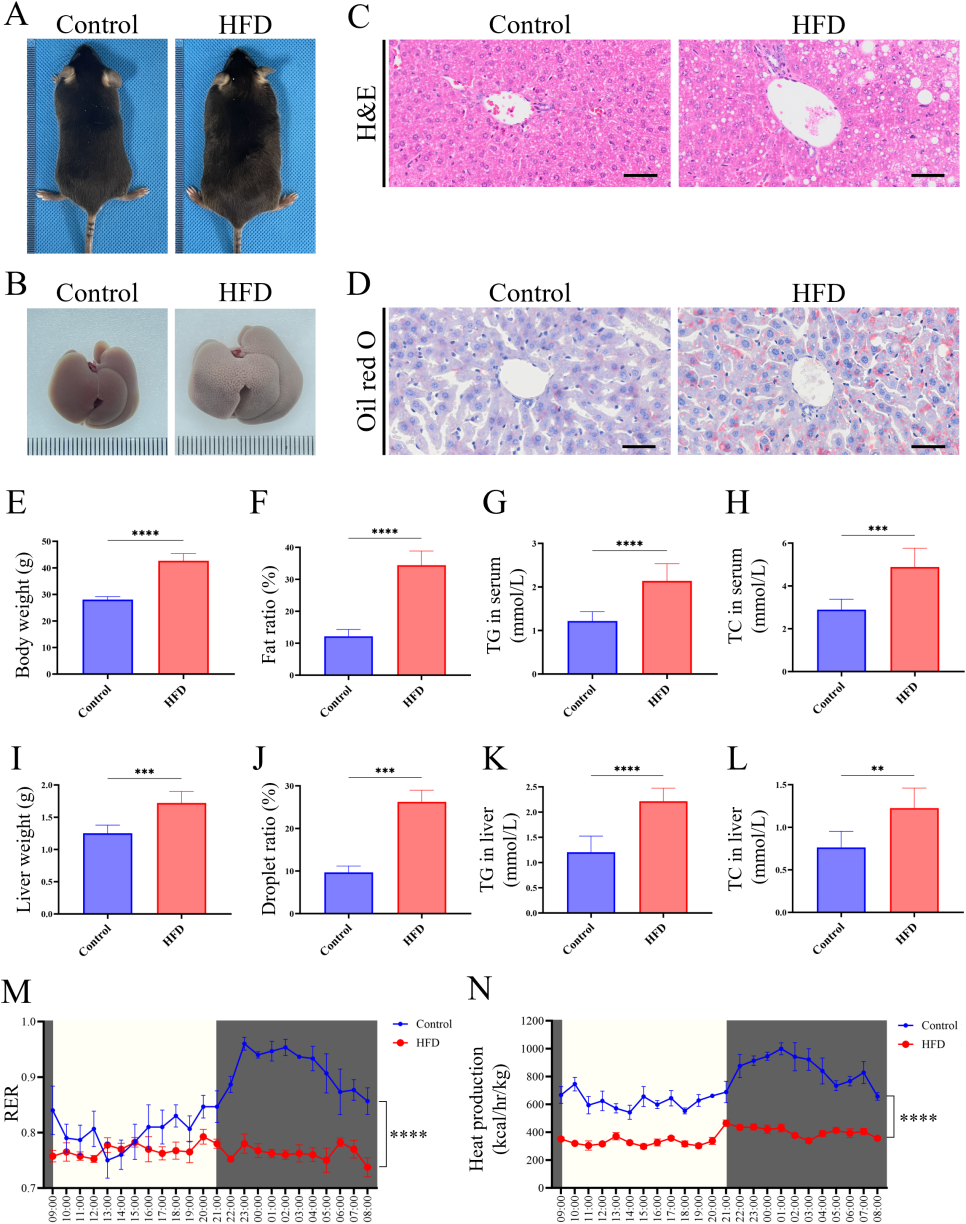
HFD-induced LMD in mice. **(A)** Morphological observation of mice; **(B)** Morphological observation of liver; **(C)** H&E staining of liver tissue sections (scale bar = 50 µm); **(D)** Oil red O staining of liver tissues (scale bar = 50 µm); **(E)** Statistical analysis of body weight; **(F)** Statistical analysis of fat ratio; **(G)** Statistical analysis of TG in serum; **(H)** Statistical analysis of TC in serum; **(I)** Statistical analysis of liver weight; **(J)** Statistical analysis of droplet ratio in liver tissue (based on the **Figure 1D**); **(K)** Statistical analysis of TG in liver tissue; **(L)** Statistical analysis of TC in liver tissue; **(M)** RER of mice in metabolic cage (non-fasted); **(N)** Heat production of mice in metabolic cage (non-fasted). **** *P* < 0.0001; *** *P* < 0.001; ** *P* < 0.01.

To begin, we investigated the phenotype of HFD-feeding mice. After 16 weeks of HFD feeding, mice in the HFD group exhibited obese appearance compared to the Control group (**Figure 1A**). The livers of HFD mice presented as an enlarged and orange peel-like appearance (**Figure 1B**). H&E and oil red O staining of liver tissue revealed vacuolar and lipid accumulation in the HFD group, whereas the Control group exhibited normal structure and lipid distribution (**Figures 1C, D**). Systemic analysis showed mice in HFD group have a higher body weight, fat ratio, TG and TC content in serum, which reflected an abnormal systemic lipid level (**Figures 1E-H**). Liver related analysis showed the HFD mice have a higher level in weight, droplet ratio, TG and TC content in liver, which are typical phenotypes of fatty liver (**Figures 1I-L**). Continuous 24-hour metabolic cage analysis showed that the RER of HFD mice was significantly lower than that of the Control group, indicating that HFD mice preferentially utilized lipids as an energy substrate, while mice of Control group primarily metabolized carbohydrates (**Figure 1M**). Additionally, the thermogenic capacity of HFD mice was significantly lower than that of the Control group (**Figure 1N**), suggesting impaired function of thermogenic-related tissues, such as brown adipose tissue. These findings together confirmed the successful establishment of LMD in mice. Given the close metabolic relationship between glucose and lipid, we simultaneously observed core indicators of carbohydrate metabolism to assess the stability of the model. GTT and ITT showed the HFD group exhibited a fluctuating high glucose level, which is insensitive to insulin (**Supplementary Figures S1A-D**). These results showed a glucose metabolic disorder and are consistent with the classic characteristics of the HFD-induced model in mice.

### LMD mice develop JOP with osteogenic impairment

**Figure 2:**
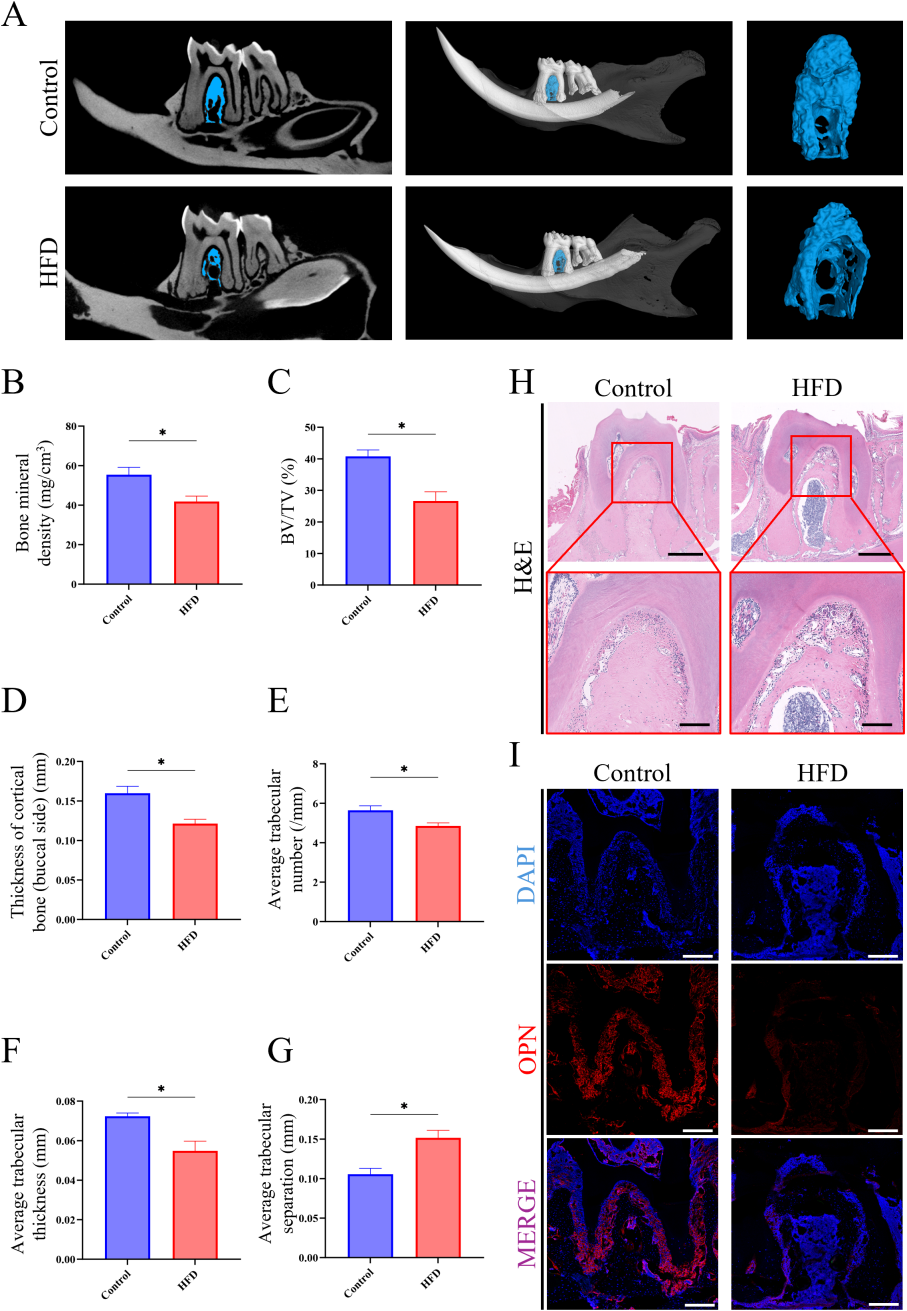
HFD-induced JOP in mice. **(A)** Micro-CT analysis of excised jawbone; **(B)** Bone density of the jawbone; **(C)** BV/TV of jawbone; **(D)** Thickness of cortical jawbone (buccal side); **(E)** Average trabecular number of jawbone; **(F)** Average trabecular thickness of jawbone; **(G)** Average trabecular separation of jawbone; **(H)** H&E staining of the molar root bifurcation area in jawbone (scale bar for up images = 500 µm; scale bar for down images = 150 µm); **(I)** Immunofluorescence staining of the molar root bifurcation area in jawbone. (red for the OPN expression, scale bar = 200 µm). *P < 0.05.

To elucidate the impact of LMD on the jawbone, a series of analyses were conducted on mice jawbone. Micro-CT imaging revealed extensive bone loss in the jawbone of HFD mice (**Figure 2A**). Further analysis showed that HFD mice exhibited significantly lower bone density, bone volume fraction (BV/TV), thickness of cortical bone (buccal side), trabecular number, and trabecular thickness compared to the Control group, while trabecular separation was higher in the HFD group (**Figures 2B-G**). Histological sections of the molar region demonstrated dense jawbone structure in Control group, whereas HFD group exhibited large areas of OP (**Figure 2H**). Immunofluorescence staining revealed significantly lower expression of OPN protein in the jawbone of the HFD group compared to the Control (**Figure 2I**). These results indicate that HFD-induced LMD disruption leads to typical osteoporosis in the jawbone. **Supplementary Data** showed a significant bone loss and typical OP in the femur samples of HFD group, which have been observed in multiple previous research and reflected the stability of this model (**Supplementary Figure S2**).

### Abnormal expression of lipid and bone metabolism-related genes are detected in the jawbone under LMD

**Figure 3:**
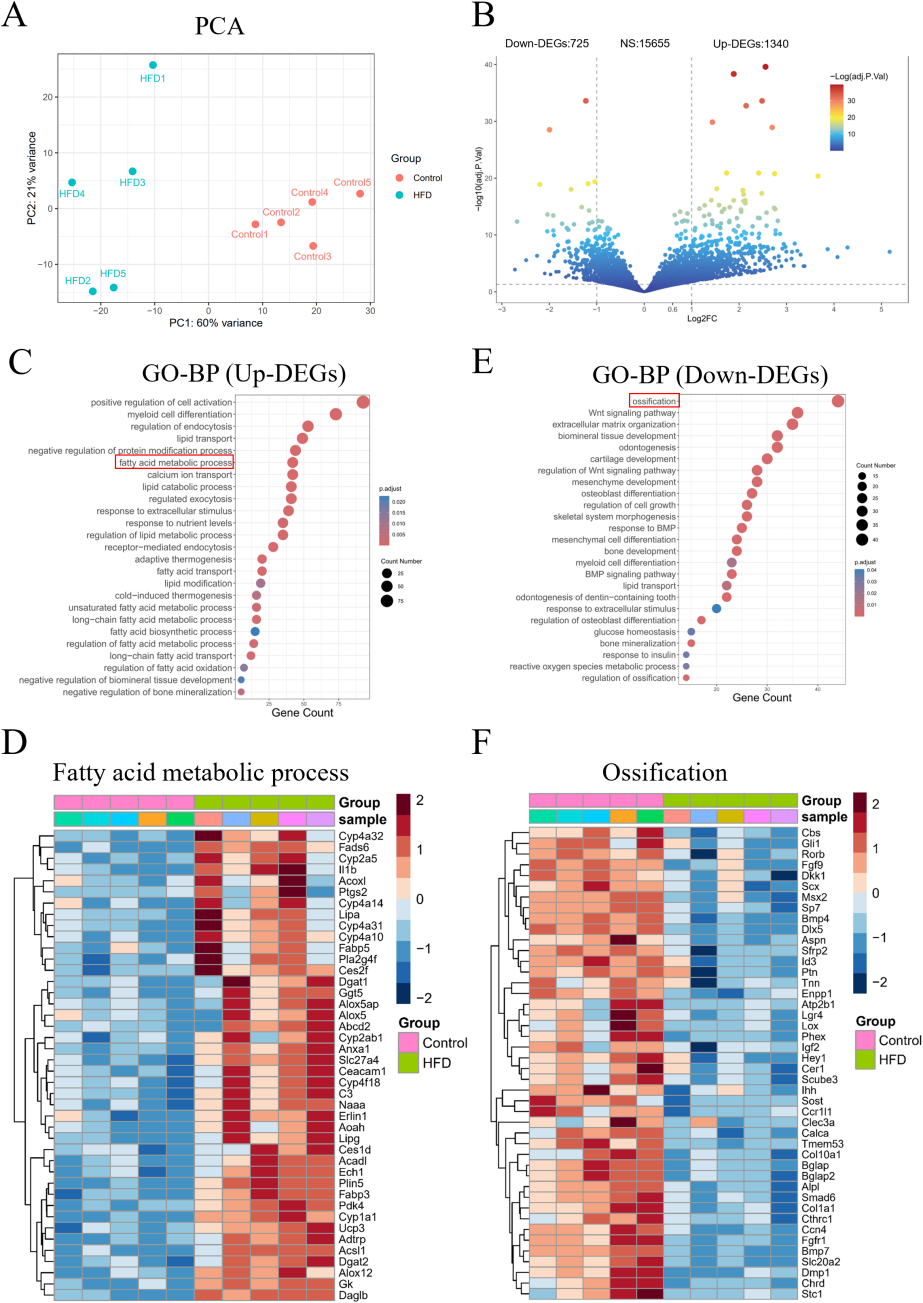
Transcriptome sequencing analysis of mouse jawbone samples. **(A)** PCA reveals the overall differences in gene expression; **(B)** Volcano plot showing the number of differentially expressed genes; **(C)** GO-BP analysis of upregulated genes; **(D)** Expression of fatty acid metabolism-related genes among upregulated genes; **(E)** GO-BP analysis of downregulated genes; **(F)** Expression of ossification-related genes among downregulated genes.

Next, we investigated at the molecular level the pathological changes of LMD-induced JOP. Transcriptomic sequencing of jawbone tissues revealed significant differences in gene expression between HFD and Control group (**Figure 3A**). Volcano plot analysis identified 1,340 upregulated and 725 downregulated genes in the HFD group, with 15,655 genes showing no significant changes (**Figure 3B**). Gene Ontology-Biological Process (GO-BP) analysis indicated that upregulated genes in the HFD group were primarily involved in lipid metabolism-related processes, such as lipid transport, fatty acid metabolic process, and lipid catabolic process (**Figure 3C**), including key genes like *Acadl*, *Fabp5*, and *Adtrp* (**Figure 3D**). Conversely, downregulated genes were mainly associated with ossification, biomineral tissue development, and osteoblast differentiation (**Figure 3E**), including critical genes like *Dkk1*, *Col1a1*, and *Alpl* (**Figure 3F**). These findings collectively demonstrate that lipid metabolism disruption leads to aberrant expression of lipid and bone metabolism-related genes in the mouse jawbone.

### BEV isolated by liberase-enhanced differential centrifugation exhibit typical EV characteristics

**Figure 4:**
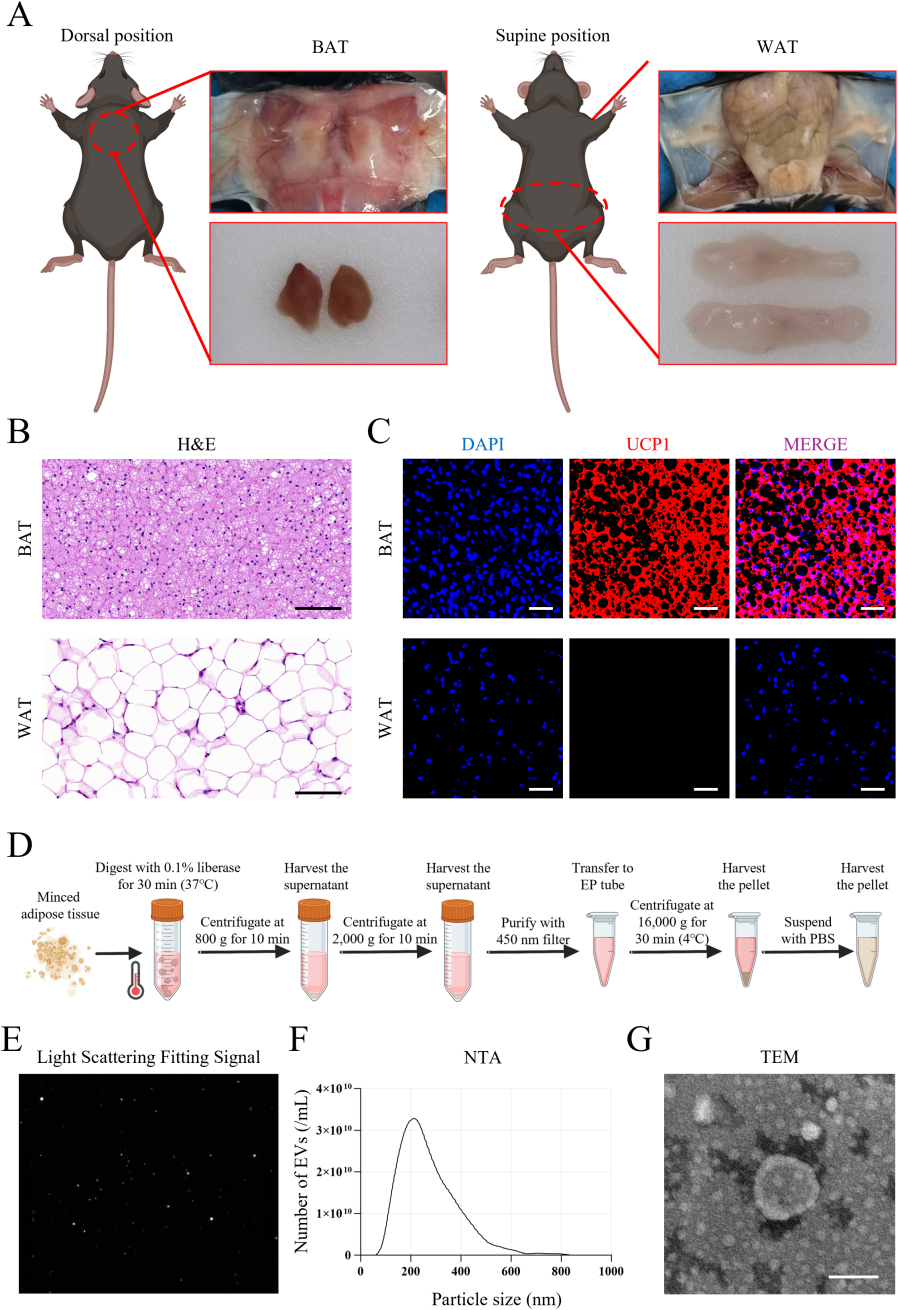
Isolation and identification of BEV. **(A)** Anatomical location and appearance of BAT and WAT; **(B)** H&E staining showed the structure of BAT and WAT (scale bar = 100 µm); **(C)** UCP1 immunofluorescence staining of BAT and WAT (red for UCP1 expression); **(D)** Brief procedure of isolation of BEV and WEV with liberase enzyme-enhanced differential centrifugation; **(E)** Light scattering fitting signal showed the dispersion and signal-to-noise ratio of BEV sample; **(F)** NTA showed the particle size distribution of BEV; **(G)** TEM showed the nanoscale morphology of BEV (scale bar = 100 nm).

We then evaluated the potential of using BEV to treat LMD and LMD-induced JOP. Anatomical dissection of mice revealed that BAT were primarily located in the subcutaneous tissue of the back, appearing as a pair of triangular tissues, while WAT were located in the subcutaneous tissue of the bilateral inguinal regions, appearing as a pair of elongated tissues (**Figure 4A**). Histological staining results showed that BAT has a dense structure with smaller cell diameters and high expression of UCP1 protein, whereas WAT has a looser structure with larger cell diameters and no detectable UCP1 protein expression (**Figures 4B, C**). The liberase-based extraction method effectively isolated BEV from adipose tissue with high dispersion and signal-to-noise ratio (**Figures 4D, E**). The average particle size of the isolated BEV was 181.0 ± 79.3 nm (**Figure 4F**), and they exhibited a distinct disc-like morphology under TEM (**Figure 4G**). Together, the successful isolation of BEV was confirmed.

### BEV are uptaken by hepatocytes, modulate the lipid metabolism, and promote an osteogenic microenvironment

**Figure 5:**
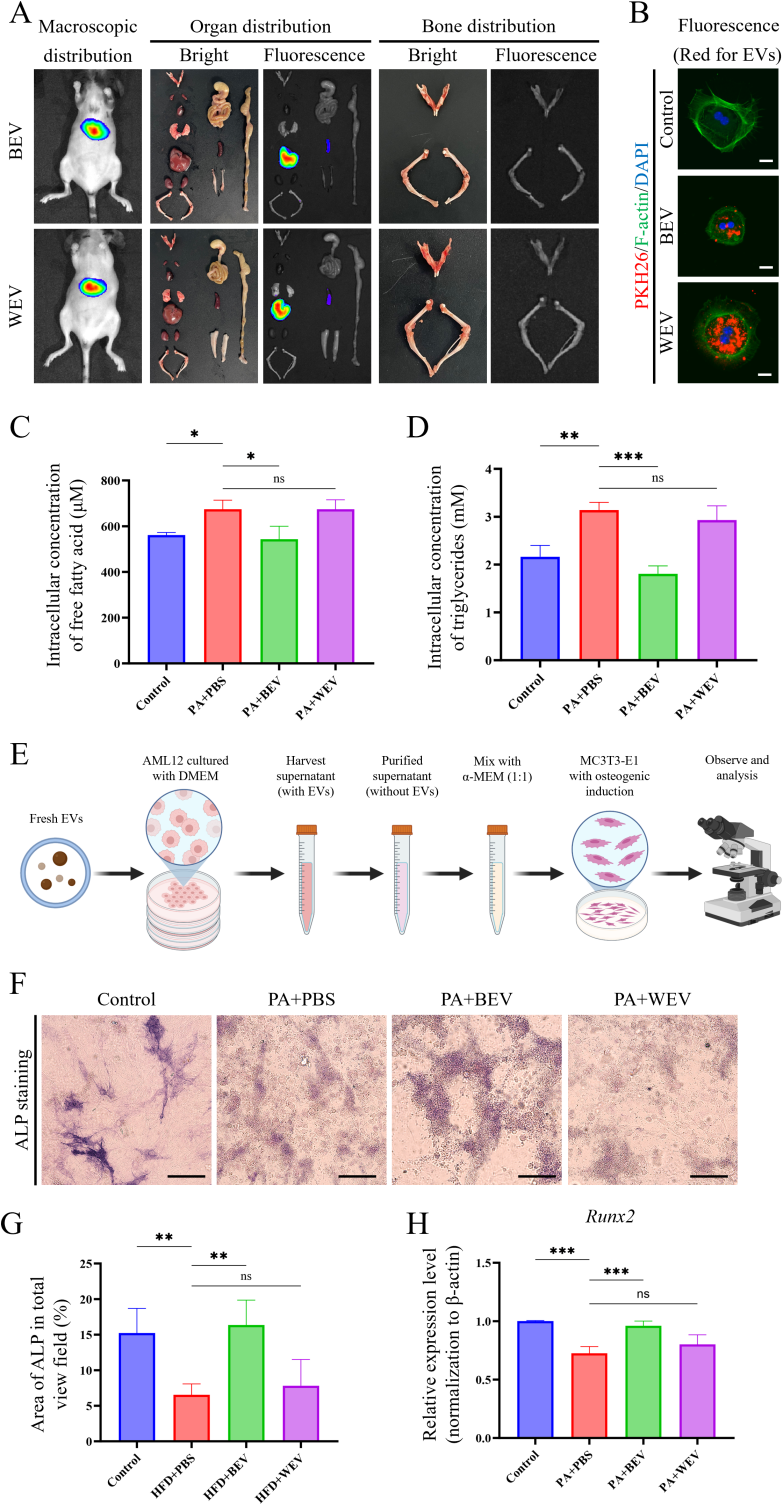
Detection of BEV biodistribution and effects on hepatocytes metabolism and osteogenic environments. **(A)** *In vivo* imaging results showing the distribution of exogenously administered BEV and WEV in mice; **(B)** Uptake of BEV and WEV (red showed the EVs labeled with PKH26) by AML12 cells; **(C)** Detection of intracellular FFA concentration in AML12 cells treated with EVs; **(D)** Detection of intracellular TG concentration in AML12 cells treated with EVs; **(E)** Brief procedure of the indirect co-culture with AML12/MC3T3-E1 cells; **(F)** ALP staining to assess the osteogenic capacity of MC3T3-E1 cells; **(G)** Statistical analysis of ALP staining results; **(H)** qRT-PCR showed *Runx2* gene expression in MC3T3-E1 cells. ***P < 0.001; **P < 0.01; *P < 0.05; ns, no significance.

To investigate the *in vivo* effects of exogenously administered BEV, we monitored their biodistribution in mice using live imaging. Results showed that fluorescence signals were primarily concentrated in the liver, followed by the spleen, with no significant signals detected in long bones or the jawbone at 24 hours post-injection of DiR-labeled BEV or WEV (**Figure 5A**). Further experiments using PKH26-labeled BEV or WEV revealed that AML12 hepatocytes efficiently internalized these EVs, which were mainly localized around the nucleus (**Figure 5B**). Under high lipid conditions simulated by PA, BEV significantly reduced intracellular TG and FFA levels in AML12 hepatocytes. Specifically, PBS-treated cells exhibited significantly higher TG and FFA levels compared to the Control group, while BEV treatment (PA+BEV) markedly decreased these levels. In contrast, WEV treatment (PA+WEV) showed no significant difference compared to PA+PBS (**Figures 5C, D**). In an indirect co-culture system of AML12 hepatocytes and MC3T3-E1 osteogenic cells under high-lipid conditions (**Figure 5E**), BEV-treated AML12 cells enhanced osteogenic activity in MC3T3-E1 cells. ALP levels in MC3T3-E1 cells were significantly lower in the PA+PBS group compared to Control, but BEV treatment (PA+BEV) significantly increased ALP levels compared to PA+PBS, while WEV treatment (PA+WEV) showed no significant difference (**Figures 5F, G**). qRT-PCR analysis revealed that *Runx2* expression was significantly lower in the PA+PBS group compared to Control, but BEV treatment (PA+BEV) significantly upregulated *Runx2* expression, with no significant difference observed in the PA+WEV group (**Figure 5H**). These findings indicated that BEV modulates the lipid metabolism in hepatocytes and indirectly promotes osteogenic activity in MC3T3-E1 cells under high-lipid conditions.

### BEV improves mitochondrial activity in AML12 hepatocytes

**Figure 6:**
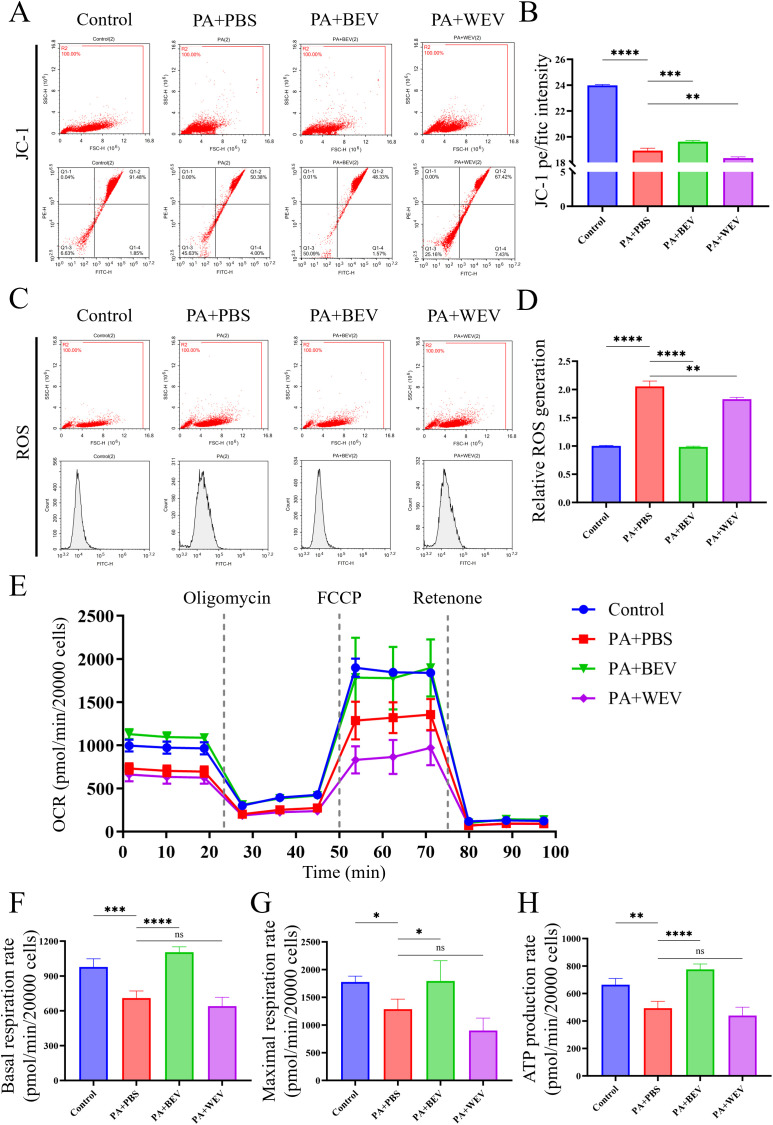
Effects of BEV on mitochondrial activity in AML12 cells. **(A)** JC-1 analysis showed the mitochondria activity of AML12 cells (flow cytometry); **(B)** Statistical analysis of JC-1 results; **(C)** ROS generation rate detection in mitochondria of AML12 cells (flow cytometry); **(D)** Statistical analysis of ROS results; **(E)** OCR detection of mitochondria (Seahorse analysis); **(F)** Basal respiration rate of mitochondria; **(G)** Maximal respiration rate of mitochondria; **(H)** ATP production rate of mitochondria. ****P < 0.0001; ***P < 0.001; **P < 0.01; *P < 0.05; ns, no significance.

Mitochondrial function tests were conducted in AML12 cells to elucidate the impact of BEV on AML12 hepatocytes (**Figure 6**). JC-1 staining revealed that the proportion of active mitochondria in AML12 cells under high lipid conditions (PA+PBS group) was significantly reduced compared to the Control group, while BEV treatment (PA+BEV group) significantly increased the proportion of active mitochondria. In contrast, the proportion of active mitochondria in the WEV-treated group (PA+WEV) further decreased below that of the PA+PBS group (**Figures 6A, B**). ROS detection showed that the ROS generation in AML12 cells under high lipid conditions (PA+PBS group) was significantly higher than in the Control group. BEV treatment (PA+BEV group) significantly reduced ROS generation, while WEV treatment (PA+WEV group) resulted in a slight decrease in ROS generation (**Figures 6C, D**). OCR analysis demonstrated that the basal respiration rate, maximal respiration rate, and ATP production rate of mitochondria in AML12 cells under high-lipid conditions (PA+PBS) were significantly lower than those in the Control group. BEV treatment (PA+BEV group) significantly improved these parameters compared to the PA+PBS group, while no significant therapeutic effect was observed in the WEV-treated group (PA+WEV) (**Figures 6E-H**). Collectively, these results indicate that high lipid conditions impair mitochondrial activity in AML12 cells, while BEV can partially restore mitochondrial function and mitigate the adverse effects of the high lipid conditions.

### BEV infusion ameliorates HFD-induced LMD

**Figure 7:**
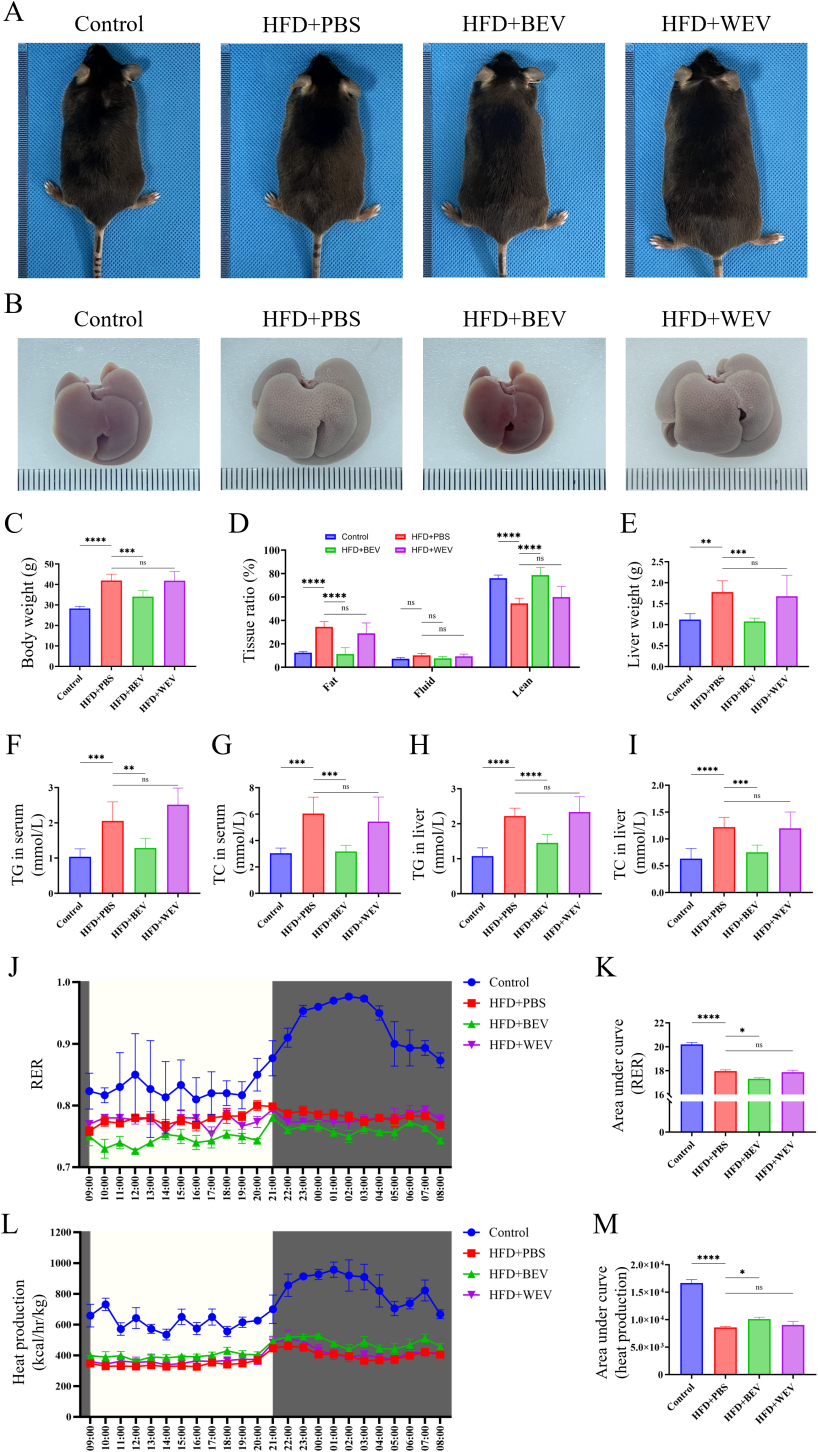
Effects of exogenous BEV administration on metabolic phenotypes in mice. **(A)** Morphological observation of mice; **(B)** Morphological observation of liver; **(C)** Statistical analysis of body weight; **(D)** Whole-body composition analysis showed the tissue ratio of fat, liquid and lean. **(E)** Statistical analysis of liver weight; **(F)** Statistical analysis of TG in serum; **(G)** Statistical analysis of TC in serum; **(H)** Statistical analysis of TG in liver; **(I)** Statistical analysis of TC in liver; **(J)** RER of mice in metabolic cage (non-fasted); **(K)** AUC of RER (based on the **J**); **(L)** Heat production of mice in metabolic cage (non-fasted); **(M)** AUC of heat production (based on the **Figures 7L**). ****P < 0.0001; ***P < 0.001; **P < 0.01; *P < 0.05; ns, no significance.

To further investigate the effects of BEV on LMD, we established an HFD-induced LMD model in mice and administered exogenous BEV intravenously as a therapeutic intervention. Results showed that BEV treatment effectively improved the obese phenotype, while the obese appearance of mice in the HFD+WEV group did not change (**Figure 7A**). Moreover, the size and appearance of the liver in the HFD+BEV group were close to those in the Control group, whereas livers in the HFD+WEV group still exhibited enlargement with an orange peel-like alteration (**Figure 7B**). Further analysis showed that HFD+BEV group exhibits lower levels than HFD+PBS and WEV-treated group in body weight, fat ratio and liver weight (**Figures 7C-E**). Biochemical assay revealed the TG and TC content in both serum and liver tissue of BEV-treated group were lower than those of HFD+PBS and HFD+WEV groups (**Figures 7F-I**). Metabolic cage analysis demonstrated that the RER of HFD+BEV group was significantly lower than that of the HFD+PBS group, while the RER of HFD+WEV group showed no significant difference compared to the HFD+PBS group (**Figures 7J, K**), which indicated that BEV treatment enhanced lipid utilization in mice with HFD. Additionally, HFD+BEV group exhibited higher thermogenic capacity than the HFD+PBS group but still lower than the Control group, while HFD+WEV group showed no significant difference in thermogenic capacity compared to the HFD+PBS group (**Figures 7L, M**). These findings suggested that exogenous administration of BEV can effectively improve obesity, and hepatic steatosis in mice through enhanced lipid utilization and increased thermogenesis.

### BEV administration improves JOP under LMD in mice

**Figure 8:**
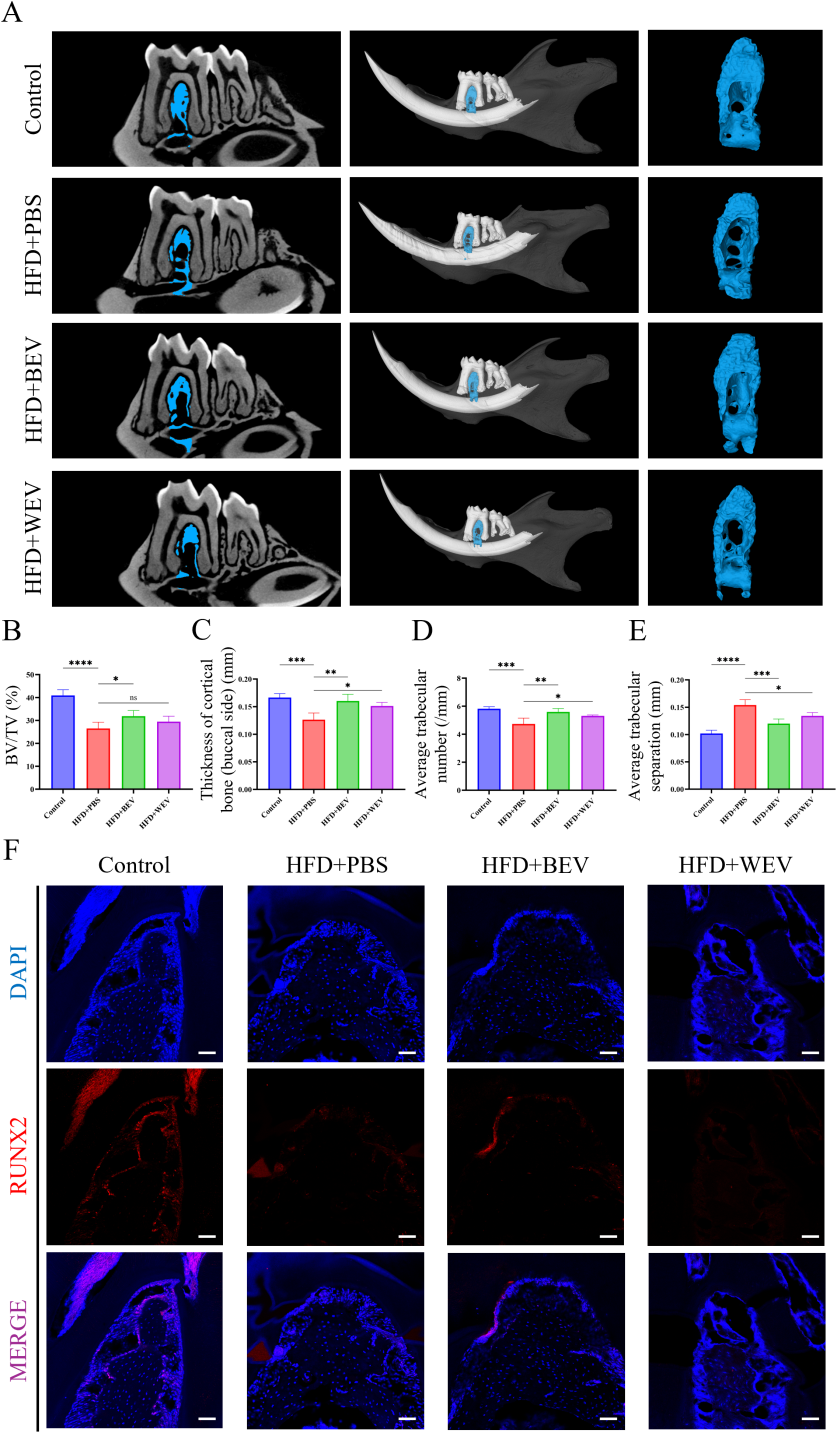
Effects of exogenous BEV administration on JOP in mice. **(A)** Micro-CT analysis of excised jawbone; **(B)** BV/TV of jawbone; **(C)** Thickness of cortical jawbone (buccal side); **(D)** Average trabecular number of jawbone; **(E)** Average trabecular separation of jawbone; **(F)** Immunofluorescence staining of the molar root bifurcation area in jawbone. (red for the RUNX2 expression, scale bar = 200 µm). ****P < 0.0001; ***P < 0.001; **P < 0.01; *P < 0.05; ns, no significance.

We further examined the changes in jawbone after BEV treatment in mice with LMD. Micro-CT analysis revealed that HFD+BEV group exhibited less bone defects in the molar region of the jawbone compared to the HFD+PBS group. Further analysis showed that HFD+BEV group showed significantly increased BV/TV, thickness of cortical bone (buccal side), and trabecular number, while decreasing trabecular separation compared to the HFD+PBS group (**Figures 8A-E**). The HFD+WEV group also showed effects on buccal cortical bone thickness, trabecular number, and trabecular separation, but to a lesser extent than HFD+BEV. Immunofluorescence staining of the molar region demonstrated significantly higher RUNX2 protein expression in the HFD+BEV group compared to the HFD+PBS group, while no significant RUNX2 expression was observed in the WEV group (**Figure 8F**). Collectively, these findings highlighted the critical role of BEV infusion to treat LMD-induced JOP in mice.

## Discussion

The complex relationship between systemic metabolic diseases and OP remains controversial (22–24). The intricate metabolic network means that both macroscopic factors (*e.g.*, increased mechanical loading due to obesity) and microscopic factors (*e.g.*, local high-lipid microenvironments in the bone marrow) induce opposing effects on the skeleton (25, 26). Additionally, the diverse structure and function of bones lead to varied responses to metabolic factors (*e.g.*, bisphosphonates reduce long-bone OP but cause jawbone necrosis) (11, 12). These factors create paradoxes and gaps in our understanding of metabolic diseases and OP. One key paradox is the “diabetic bone paradox”: while animal studies show bone loss in long-bone due to diabetes, clinical observations often report that diabetic patients keep a normal or higher bone density (although with a higher fracture risk) (27, 28). This suggests low consistency between human metabolic diseases and long-bone density. The latest explanation is that increased body weight in diabetic patients exerts additional mechanical loading on long-bones, partially offsetting the cause of bone loss (29). Meanwhile, high-glucose environments lead to advanced glycation end-products (AGEs) accumulation in the bone marrow, causing abnormal extracellular matrix structure and reduced bone toughness, thereby increasing fracture risk (30). Differences in posture, exercise habits, and load bearing between animals and humans also limit the translatability of animal studies to clinical phenomena. Notably, compared to OP in long bones, JOP under metabolic disorders is underexplored. Inducing LMD *via* HFD in animal models to simulate periodontal diseases (*e.g.*, periodontitis) shows good consistency with clinical observation (7, 31). Our study found that a 16-week HFD induced LMD and JOP in mice, with transcriptomic sequencing revealing downregulated osteogenic genes. *In vitro* experiments also showed that high-lipid conditions significantly inhibited osteogenic activity. These results indicated that JOP under LMD is highly correlated with clinical observations, thereby holding significant clinical relevance.

Adipose tissue has been re-realized over the past few years around its role in systemic metabolism, especially the two types of thermogenic adipose tissue (BAT and BeAT) (15). BeAT originates from WAT through a “browning” process under specific conditions (cold exposure, starving, fasting, and hormone stimulus) (14). However, BeAT can revert to WAT once the stimulating factors are removed, and this instability largely restricts its further application. In contrast, BAT exhibits a more robust and stable capacity for metabolic regulation in the context of direct transplantation or EVs extraction. BEV, as vesicular bodies secreted by brown adipocytes, have been demonstrated to exert functions mimicking their parental cells (17, 18). However, the costly and low-yield extraction of BEV from large-scale cultures limits their clinical application, making increased yield an urgent challenge (32). Tissue-derived EVs extraction, as a novel vesicle isolation technique, emphasizes the direct extraction of EVs from tissues while bypassing the cell culture step (33, 34). This method simplifies extraction and enhances yield, showing promise for clinical applications. In this study, we directly extracted EVs from BAT and verified their metabolic regulatory effects. *In vivo* experiment results showed that BEV significantly improved HFD-induced LMD, which is due to the BEV-mediated thermogenic and utilization of lipids. In cellular experiments, we found that BEV does not accumulate in the jawbone but enhances mitochondrial activity in hepatocytes, thereby creating an osteogenic-favor microenvironment. For the first time, we demonstrated the dual effects of BEV on both LMD and JOP, highlighting its broad potential for clinical applications. On the other hand, given that WEV derived from WAT (non-thermogenic adipose), it shares similarities with BEV while keeping specific differences in thermogenic capacity compared to BEV (35, 36). Moreover, considering the extensive realization has been well established on WEV, it serves as an ideal negative control in this study.

Hepatocytes, the primary cells involved in lipid metabolism, depend significantly on the activity of mitochondria (37). Enclosed by a double-membrane structure and containing numerous metabolic enzymes (*e.g.*, fatty acid dehydrogenase, carnitine palmitoyl transferase), mitochondria can break down fatty acids for energy production, generate ketone bodies, and supply acetyl-CoA as a precursor for *de novo* lipid synthesis (38, 39). Under normal conditions, the metabolic rates of various substances within mitochondria are dynamically stable, forming mitochondrial homeostasis (40). When mitochondrial function is compromised, the body is capable of preserving mitochondrial homeostasis through several mechanisms, including mitophagy, mitochondrial fission and fusion, as well as mitochondrial transfer (41, 42). Recent studies have shown that EVs secreted by cells can contain mitochondrial-derived membranes and protein components, which can participate in host mitochondrial activities upon uptake, thereby supporting normal metabolic functions (41). This EVs-mediated mitochondrial transfer mechanism between same or different cells has been demonstrated as an important part in keeping balanced mitochondrial function (43). Although direct mitochondrial content exchange between BEV and hepatocytes has not been reported *in vivo*, this study indirectly observed this phenomenon through exogenous administration of BEV. *In vitro* experiments showed that the AML12 cells exhibited impaired mitochondrial function in high-lipid conditions, but the mitochondrial function was significantly restored with the uptakes of BEV. As an additional benefit of BEV treatment, AML12 cells facilitated the formation of an osteogenic-favor microenvironment that enhanced osteogenic activity of MC3T3-E1 cells. This result further validates that exogenous EVs administration to simulate mitochondrial transfer is a promising direction for treating mitochondrial damage-related diseases in the field of biotherapy.

However, there are still some noteworthy phenomena that need to be explored. For example, given the equal concentration of BEV and WEV (both labelled with PKH26) before uptake by AML12, the BEV showed a limited accumulation in cells, which is significantly lower than WEV (**Figure 5B**). Moreover, all the following experiments showed the BEV possesses a stronger regulatory capability despite limited uptake. This may be due to BEV playing its positive role relying on the cargo or membrane materials distinguished from WEV rather than simply dose-dependent effects. Based on this hypothesis, a novel tissue-engineering modification strategy that enhances the targeting or accumulating capacity of BEV in cells is expected to further increase its treatment benefit. Although WEV did not show significant therapeutic effects in most experiments in this study, a slight decrease in ROS was still observed in the PA+WEV group (**Figure 6D**). Moreover, the HFD+WEV group exhibited mild improvements in some micro-CT indicators (*e.g.*, thickness of cortical bone, trabecular number, and trabecular separation) in animal experiments (**Figures 8C-E**). These phenomena may be due to the modest restoration of WEV on hepatocyte metabolic function by supplementing essential mitochondrial components. This further suggests that the therapeutic effects of BEV are a comprehensive result of multiple biological effects beyond the BEV-mediated thermogenic activity. However, as one of the key distinctions between BAT and WAT, the direct effects of UCP1 in this process are still unclear (44). Dividing BEV into UCP1^+^ and UCP1^-^ subpopulations using nano-flow sorting technology and observing their respective effects will be the focus of our future research to answer this question.

## Conclusion

This study establishes LMD as a crucial driver factor in JOP and identifies BEV-mediated mitochondrial transferring in hepatocytes as a therapeutic strategy for LMD-related JOP.

## Data Availability

The raw sequencing data have been deposited in the Genome Sequence Archive (GSA) of China National Center for Bioinformation under accession number CRA024686 (https://ngdc.cncb.ac.cn/gsa/).

## Supplementary material

Supplementary Figure 1 HFD-induced glucose metabolic disorder in mice. **(A)** Glucose tolerance test (GTT); **(B)** Area under curve based on **Supplementary Figures S1A** **(C)** Insulin tolerance test (ITT); **(D)** Area under curve based on **Supplementary Figures S1C**. **** *P* < 0.0001; *** *P* < 0.001.

Supplementary Figure 2 Micro-CT analysis of femur. **(A)** Micro-CT analysis of excised femur; **(B)** Bone density of the femur; **(C)** BV/TV of femur; **(D)** Thickness of cortical bone; **(E)** Average trabecular number; **(F)** Average trabecular thickness; **(G)** Average trabecular separation. **** *P* < 0.0001; *** *P* < 0.001; * *P* < 0.05; ns = no significance.

Supplementary Figure 3 Procedure for establishing the LMD model to analyze the effects of BEV on metabolic phenotypes in mice.
