# Supplementary figures and images for: Brown adipose tissue-derived extracellular vesicles regulate hepatocyte mitochondrial activity to alleviate high-fat diet-induced jawbone osteoporosis in mice
Source: Front Endocrinol (Lausanne). 2025 Apr 24;16:1583408. doi: 10.3389/fendo.2025.1583408 (PMC12058480; doi:10.3389/fendo.2025.1583408)

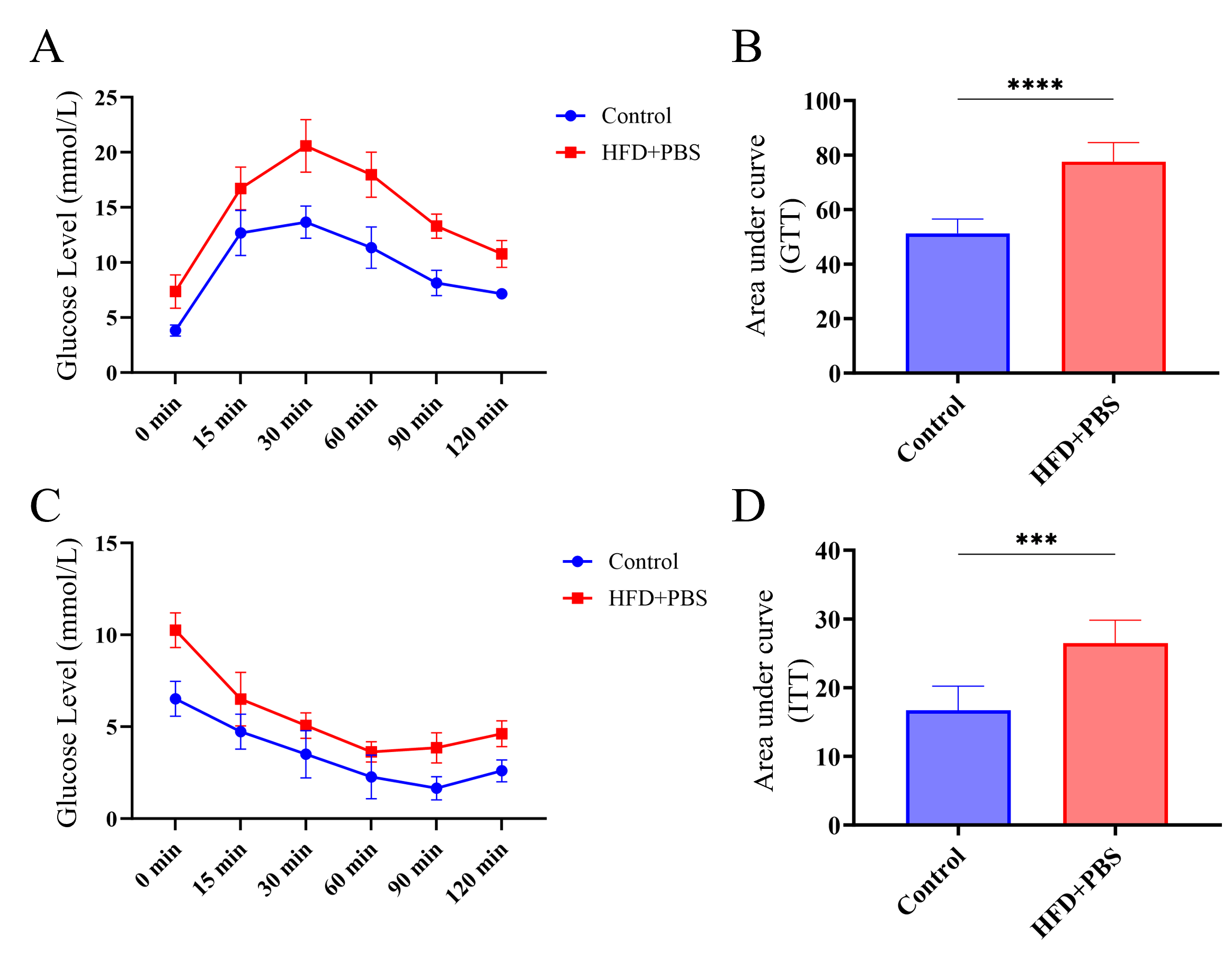

Supplement: Supplementary Figure 1 — HFD-induced glucose metabolic disorder in mice. (A) Glucose tolerance test (GTT); (B) Area under curve based on Supplementary Figures S1A (C) Insulin tolerance test (ITT); (D) Area under curve based on Supplementary Figures S1C . **** P < 0.0001; *** P < 0.001. [file Image1.tif]

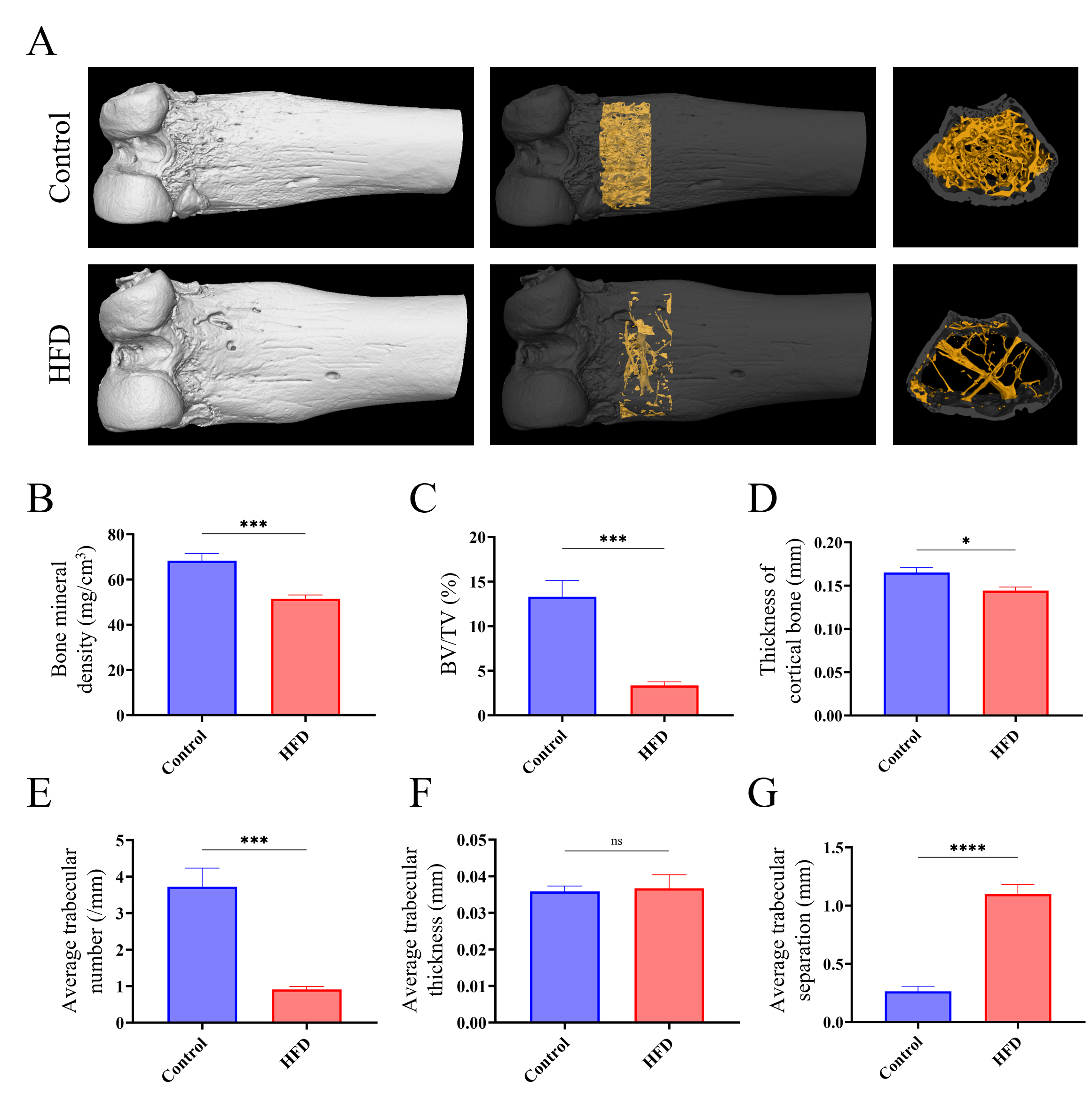

Supplement: Supplementary Figure 2 — Micro-CT analysis of femur. (A) Micro-CT analysis of excised femur; (B) Bone density of the femur; (C) BV/TV of femur; (D) Thickness of cortical bone; (E) Average trabecular number; (F) Average trabecular thickness; (G) Average trabecular separation. **** P < 0.0001; *** P < 0.001; * P < 0.05; ns = no significance. [file Image2.tif]

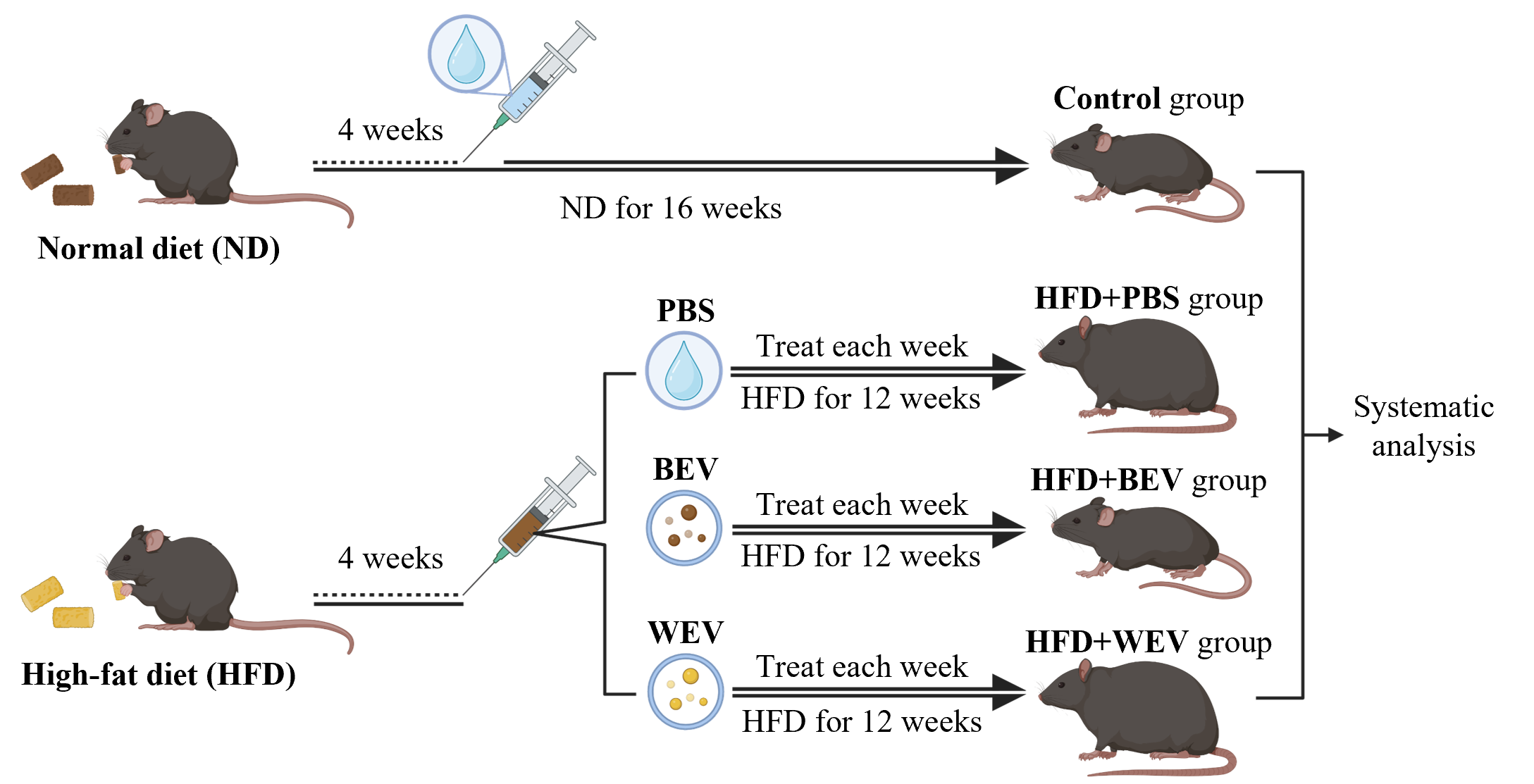

Supplement: Supplementary Figure 3 — Procedure for establishing the LMD model to analyze the effects of BEV on metabolic phenotypes in mice. [file Image3.tif]
